# Numerical Study for the Performance of Viscoelastic Fluids on Displacing Oil Based on the Fractional-Order Maxwell Model

**DOI:** 10.3390/polym14245381

**Published:** 2022-12-08

**Authors:** Jingting Huang, Liqiong Chen, Shuxuan Li, Jinghang Guo, Yuanyuan Li

**Affiliations:** 1 State Key Laboratory of Oil & Gas Reservoir Geology and Exploitation, Southwest Petroleum University, Chengdu 610500, China; 2 North China Oil and Gas Company, Sinopec, Zhengzhou 712034, China; 3 The Tenth Oil Production Team in the North of the Sixth Operation Area of No. 1 Oil Production Plant, Daqing Oilfield, Daqing 163000, China

**Keywords:** non-Newtonian fluid, fractional-order Maxwell model, displacement efficiency, polymer flooding

## Abstract

In the study of polymer flooding, researchers usually ignore the genetic stress properties of viscoelastic fluids. In this paper, we investigate the process of viscoelastic fluid flooding the remaining oil in the dead end. This work uses the fractional-order Maxwell in the traditional momentum equation. Furthermore, a semi-analytic solution of the flow control equation for fractional-order viscoelastic fluids is derived, and the oil-repelling process of viscoelastic fluids is simulated by a secondary development of OpenFOAM. The results show that velocity fractional-order derivative α significantly affects polymer solution characteristics, and increasing the elasticity of the fluid can significantly improve the oil repelling efficiency. Compared to the Newtonian fluid flow model, the fractional order derivative a and relaxation time b in the two-parameter instanton equation can accurately characterize the degree of elasticity of the fluid. The smaller the a, the more elastic the fluid is and the higher the oil-repelling efficiency. The larger the b, the less elastic the fluid is and the lower the cancellation efficiency. Moreover, the disturbance of the polymer solution to the dead end is divided into two elastic perturbation areas. The stronger the elasticity of the polymer solution, the higher the peak value of the area in the dead end and the higher the final oil displacement efficiency.

## 1. Introduction

In the structure of the global oil resources reserves, 40% is viscous oil and ultra-heavy oil. However, the major viscous oilfields enter the late stage of development in most countries. Long-term water injection makes the remaining oil distribution in the reservoir of these viscous oilfields very dispersed. Water flooding mining can no longer meet the demand for enhanced recovery from these waterflooded reservoirs [1,2,3,4]. Tertiary oil recovery provides new ideas to further improve recovery from waterflooded reservoirs. Among them, polymer and composite oil flooding have been widely applied in China, which is the main recovery enhancement technology for viscous oilfields. Actual production results show that polymer and composite oil flooding technology can increase the recovery rate of oilfields with high water cuts by 10% on average [5,6]. However, in the face of the huge and growing demand for oil resources, the 10% increase in recovery rate will not solve the gap in resource demand. Therefore, how to improve the recovery rate of polymer and composite flooding technology has become a hot issue in recent years.

The major factor for enhanced oil recovery from polymer flooding is changing the seepage characterization of water and enhanced volumetric sweep efficiency by using polymer [7,8,9]. Many researchers have investigated the effect of flow characterization of polymer solution on the polymer flooding enhanced oil recovery rate. The flow characterization can be divided into two parts: elasticity and viscosity. Numerous indoor tests have shown that the elasticity of the polymer solution contributes more to enhanced oil recovery rate than viscosity. Wang et al. carried out core displacement experiments of elastic solution and viscoelastic solution and found that the displacement efficiency obtained by the viscoelastic fluid is 6% higher than that obtained by elastic fluid, which proves that viscoelasticity is the main mechanism of enhancing oil recovery [10,11]. In addition, through core and glass etching experiments, Xie and Hosseini also found that during the process of polymer flooding, the front end of the polymer solution spread more slowly, and the spread scope was larger [12,13]. Through the research results of the above scholars, it can be found that the more elastic the polymer solution is, the more it can flood the remaining oil out of the dead space in the rock pore network. Many experimental results observing the flow pattern of polymer solution in micropores have shown that elastic polymers flow in micropores with irregular disturbances, which is due to elastic turbulence caused by polymers in solution. Groisman, Poole, Arratia, et al., through experimental research, found that the viscous fluid containing a small amount of high molecular weight polymer solution produces an irregular flow structure similar to turbulence at low Re number [14,15,16,17]. Mitchell and Howe used MRI experiments to demonstrate the effect of polymer-specific elastic turbulent flow on enhanced oil recovery [18,19].

The law of viscoelastic polymer flooding is studied in two types of experiment and numerical simulation. In recent years, the grid model has mainly been used to study the fluid–fluid repulsion process in microporous channels; the advantage of this method is that it can establish a flow condition close to the real formation and the experimental results are more reliable [20,21,22,23]. However, to accurately capture the flow within the microporous channels, techniques such as CT scans, X-rays, and MRI are required; this makes experimental studies very expensive and time-consuming, and it is difficult to obtain a dynamic view. Numerical simulation studies can overcome this drawback. The most important thing in numerical simulation is that the intrinsic model can accurately reflect the flow characteristics of non-Newtonian fluids. In previous studies, polymer solutions are simplified to a Newtonian fluid or used the integer-order non-Newtonian model to describe the constitutive relation [24,25,26]. Although these models can respond to some extent to the rheological properties of non-Newtonian fluids. However, it is not possible to accurately characterize the genetic and stress relaxation properties of complex polymeric fluids such as flooding fluid.

In recent years, with the development of fractional-order theory and computational methods, researchers have combined fractional-order theory with fluid mechanics to establish the fractional-order constitutive equations of fluids, such as the fractional-order Maxwell model, fractional-order Jeffreys model, and fractional-order O-B model [27,28,29,30]. Due to the unique physical significance of fractional order, it can accurately describe the viscoelastic flow characteristics of non-Newtonian fluids; thus, it is widely used with complex physical problems, such as the plane surface-flow [31], flow on coaxial cylinders [32], and Marangoni [33].

Research on fractional-order fluids has mainly focused on single-phase flow, and the problem of microscale multiphase flow in polymer flooding processes has not been solved. In this study, we developed a fractional-order semi-analytical transient model for calculating the velocity and pressure of polymer solution. The model divides the shear stress during the flow of polymer solution into two stress fields, Newtonian, and fractional-order non-Newtonian. The numerical solution is calculated for the Newtonian stress field, and the analytical solution for the fractional-order non-Newtonian stress field is obtained using the Laplace variation. We can predict the efficiency of polymer flood oil based on the model that performed a secondary development of the interFoam incompressible two-phase flow solver in OpenFOAM.

## 2. Physical and Mathematical Models

### 2.1. Physical Model

**Figure 1:**
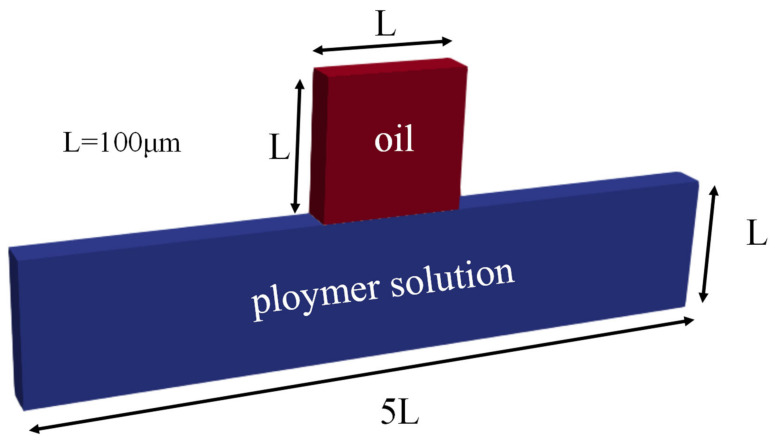
Physical model of the dead-end capillary.

In this paper, we investigated the dead-end capillary tube that is a representative residual oil capillary structure such as in Figure 1. The length of the capillary is 500 microns, and in the middle of the capillary, there is a dead-end capillary with a depth of 100 microns, which is filled with residual oil.

**Figure 2:**
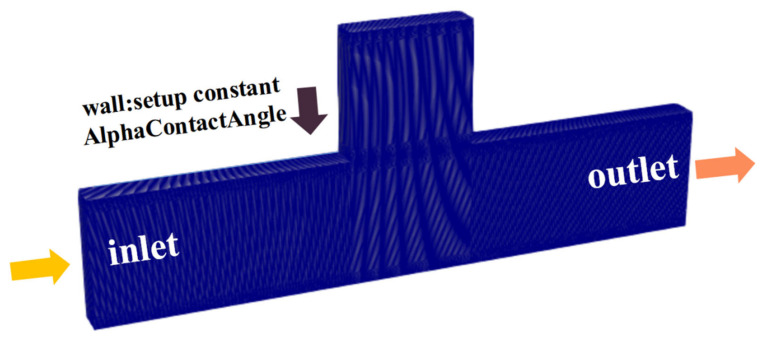
Numerical mesh model of the dead-end capillary.

Figure 2 is the numerical mesh model based on the physical model, which contains 240,000 rectangular cells. The left side is the inlet and the right side is the outlet; the rest of the boundary is the wall. Particularly, the wall adhesion effect is taken into consideration by defining a contact angle (oil to water: 45°) at the wall such as Equation (1), which is aimed to simulate the wall effect of oil in contact with rock [34]:

(1) $$\hat{N}=\hat{N_{w}}cos\theta_{w}+\hat{M_{w}}sin\theta_{w}$$

### 2.2. Governing Equation of Fluid

When the above fluids are incompressible, the continuity equation is

(2) ∇⋅ U=0

For the incompatible polynomial system momentum equation, which considers gravity and source terms:

(3) $$\frac{\partial \rho UU}{\partial t}+\nabla \cdot (\rho UU)=-\nabla p+\rho g+F_{f}+\nabla \cdot \tau$$

When the two-phase fluid interface flow is in equilibrium, $F_{f}=\Delta P_{f}$ represents the interfacial tension, which is equal to the pressure difference at the two-phase fluid interface. The interfacial tension in this equilibrium condition is treated by the CFS method [35]:

(4) $$F_{f}=\Delta P_{f}=\tau \kappa \Delta \alpha \;\kappa =-\nabla \cdot \;n$$

According to the treatment of the interfacial tension, the phase volume fraction of the two-phase fluid in the calculated region is unknown; therefore, the equation for the phase fraction is also needed. Applying the method proposed better for the phase volume fraction [36],

(5) $$\frac{\partial \alpha }{\partial t}+\nabla \cdot (\alpha U)+\nabla \cdot (\alpha (1-\alpha )c|U|\frac{\Delta \alpha }{\left|\Delta \alpha \right|})=0$$

where *c* denotes the controllable compression factor, when there is no compression effect. The larger the compression effect, the faster and more pronounced the compression effect.

### 2.3. The Constitutive Equation of Viscoelastic Fluid

The relationship between the shear stress and shear rate is defined as the constitutive equation of fluid flow. When the fluid is considered a Newtonian fluid, the shear stress term τ in Equation (3) is τ=2μ∇U. However, this relationship is not suitable for fluid that is considered non-Newtonian. For polymer solutions, the shear stress can be divided into two parts: the stress tensor of the solvent $\tau_{w}$ and the solute $\tau_{p}$, which is:

(6) $$\tau =\alpha_{w}\tau_{w}+\alpha_{p}\tau_{p}$$

where the aforementioned represent the mass fraction of the solvent and solute in the polymer solution, respectively. $\tau_{w}$ is considered Newtonian and is considered non-Newtonian. In this paper, the fractional-order Maxwell model is used to describe $\tau_{p}$

(7) 1+λ1aD0Ctaτ=μdSdt

where ${}_{a}^{C}D_{t}^{a}$ is the fractional-order differential defined by using Caputo:

(8) D0Ctaf(t)=1Γ(n−a)∫t0fn(ξ)dξ(t−ξ)a−n+1

The gamma Function in Equation (8) is defined as

(9) $$\Gamma (z)=\int_{\infty }^{t}\exp^{-t}t^{z-1}dt$$

### 2.4. Numerical Algorithms

Above governing and constitutive equations of polymer solution, the momentum equation is:

(10) $$\frac{\partial \rho UU}{\partial t}+\nabla \cdot (\rho UU)=-\nabla p+\rho g+F_{f}+\nabla \cdot \tau_{w}+\nabla \cdot \tau_{p}$$

The time-degenerate nature of fractional-order integrals leads to numerical discretization that cannot be adopted by a step-by-step method for integer-order solution derivatives. There are many numerical solution of fractional-order integral operators ${}_{0}^{C}D_{t}^{a}$ [37,38,39]. The L1 discrete format is a common difference format for momentum equations containing time fractional-order differentiation [39]. Based on a discrete format, researchers have achieved certain results in the study of the effect of viscoelasticity for non-Newtonian fluids on their flow laws. Equation (11) is the fractional-order Maxwell fluid boundary layer flow equation that bases on the L1 discrete format derived by J.H Zhao.

(11) $$\frac{\partial u}{\partial t}+u\frac{\partial u}{\partial x}+v\frac{\partial u}{\partial y}+\underset{fractional-ordertimeandconvectionterms}{\underset{︸}{\lambda_{1}^{\alpha }\frac{\partial^{\alpha +1}u}{\partial t^{\alpha +1}}+\lambda_{1}^{\alpha }\frac{\partial^{\alpha }}{\partial t^{\alpha }}\left(u\frac{\partial u}{\partial x}\right)+\lambda_{1}^{\alpha }\frac{\partial^{\alpha }}{\partial t^{\alpha }}\left(v\frac{\partial u}{\partial y}\right)}}=\frac{\partial^{2}u}{\partial y^{2}}+\lambda_{1}^{\alpha }\frac{\partial^{\alpha }\theta }{\partial t^{\alpha }}+\theta$$

After replacing the intrinsic model with the fractional-order Maxwell model, the momentum equation is additionally increased with a fractional-order time and convection term. Equation (12) is the result of discretizing and dimensionless processing of Equation (11)

$$-\left(1+r_{1}\right)\frac{\Delta t}{\Delta x}u_{i,j}^{k-1}u_{i-1,j}^{k}-\left[\left(1+r_{1}\right)\frac{\Delta t}{\Delta y}v_{i,j}^{k-1}+r_{2}\right]u_{i,j-1}^{k}$$

(12) $$+\left[\left(1+r_{1}\right)\left(1+\frac{\Delta t}{\Delta x}u_{i,j}^{k-1}\right)-\left(1+r_{1}\right)\frac{\Delta t}{\Delta y}v_{i,j}^{k-1}+2r_{2}\right]u_{i,j}^{k}-r_{2}u_{i,j+1}^{k}$$

$$=r_{2}u_{i,j-1}^{k-1}+\left(1+r_{1}-2r_{2}\right)u_{i,j}^{k-1}+r_{2}u_{i,j+1}^{k-1}+r_{1}A_{1}+\frac{\Delta t}{\Delta x}r_{1}A_{2}+\frac{\Delta t}{\Delta y}r_{1}A_{3}$$

$$+\Delta {\mathrm{tr}}_{1}A_{4}+\frac{\Delta t\left(\theta_{i,j}^{k-1}+\theta_{i,j}^{k}\right)}{2}$$

(13) $$v_{i,j}^{k}=v_{i,j-1}^{k}+v_{i,j-1}^{k-1}-v_{i,j}^{k-1}+\frac{\Delta y}{2\Delta x}\left(\begin{array}{l} u_{i-1,j-1}^{k-1}-u_{i,j-1}^{k-1}+u_{i-1,j}^{k-1}-u_{i,j}^{k-1}\\ +u_{i-1,j-1}^{k}-u_{i,j-1}^{k}+u_{i-1,j}^{k}-u_{i,j}^{k} \end{array}\right)$$

**Figure 3:**
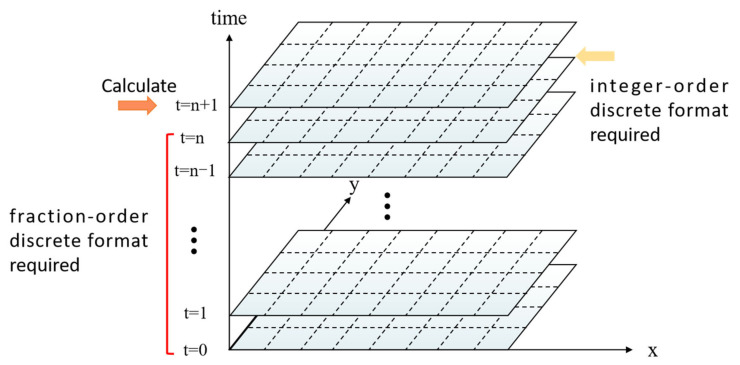
Fractional-order versus integer-order temporal step advance calculation.

**Figure 4:**
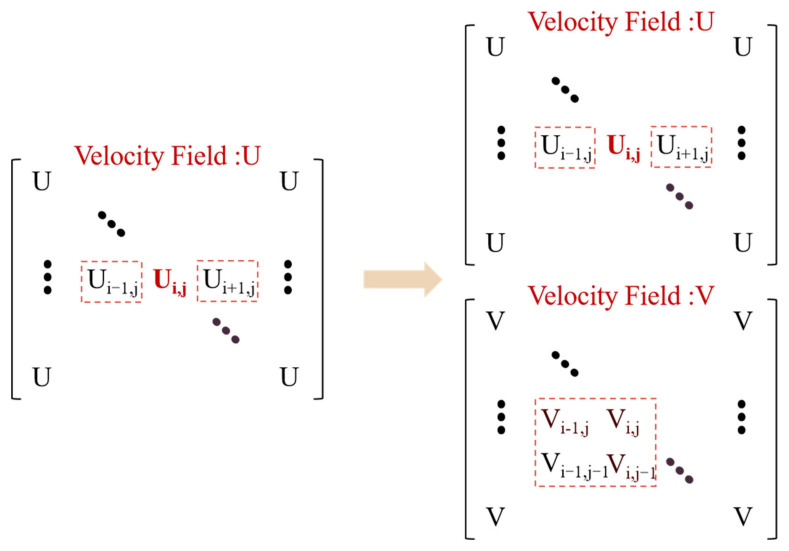
Fractional-order versus integer-order special step advance calculation.

As shown in Figure 3 and Figure 4, the momentum equation containing fractional-order terms requires a large change in the physical field at each temporal and special step compared to the integer-order. In terms of a temporal step progression, the fractional-order calculation requires extraction of the physical field for all the previous time travels, while the integer-order generally requires only the last one or two temporal steps. As the temporal step advances, the number of physical fields to be computed at the next time will become larger and larger, greatly increasing the computational overhead. In terms of special step progression, the velocity components U and V, in both the x and y directions affect the velocity U when calculating the fractional order momentum equation. This coupled solution of multiple velocity fields will increase the nonlinearity of the matrix, making it not easy to obtain stable and accurate calculation results in the presence of drastic changes in physical fields at the interface of two-phase intersections such as multiphase flow.

Based on the physical problem investigated in this paper, we proposed an approximate method to solve the computational problem of fractional order momentum equations in complex flows. The Reynolds number of the polymer solution in the capillary during the flooding processing is generally on the order of $10e^{-3}$. Several assumptions are made under the physical model we have developed:

(1) The fluid flow in a certain time interval can be regarded as a completely steady state. Therefore, under the physical model we developed in Section 2.1, the strain of the fluid during half the length of the flow through the capillary is assumed to be constant;
(2) Polymers are completely dissolved in water and uniformly distributed in the flow field;
(3) The flow state of the polymer is exactly the same as that of water; i.e., the velocity distribution of both is exactly the same.

Therefore, by Laplace transformation, Equation (7) becomes

(14) $$s^{\alpha }\bar{\varepsilon }=\frac{1}{E}s^{\alpha }\bar{\tau }+\frac{1}{\eta }\bar{\tau }$$

Rectification to obtain relaxation modulus G(t):

(15) $$G(s)=\frac{\bar{\tau }}{s\bar{\varepsilon }}=\frac{s^{\alpha -1}}{\frac{s^{\alpha }}{E}+\frac{1}{\eta }}$$

Using Laplace inverse transformation in Equation (14),

(16) $$G(t)=L^{1}\left[E\sum_{k=0}^{\infty }{\left(-1\right)}^{k}s^{-\alpha k-1}\right]=EH_{1,2}^{1,1}\left.\left[\frac{Et^{\alpha }}{\eta }\right.\left|{}_{\left(0,1);(0.1\right)}^{\left(0,1\right)}\right|\right]$$

where $H_{1,2}^{1,1}$ is *H*-Fox function, which is defined as:

(17) $$\sum_{n=0}^{\infty }\frac{{\left(-z\right)}^{n}\prod_{i=1}^{p}\Gamma (a_{i}+A_{i}n)}{n!\prod_{i=1}^{q}\Gamma (b_{i}+B_{i}n)}=H_{P,Q+1}^{1,P}\left[z\left|{}_{\left(0,1);(1-b_{q},B_{q}\right)}^{\left(1-\alpha_{p},A_{p}\right)}\right|\right]$$

Converting Equation (15) into an M-L function:

(18) $$H_{1,2}^{1,1}\left[\frac{Et^{\alpha }}{\eta }|\;\left|{}_{\left(0.1);(0,1\right)}^{\left(0,1\right)}\right|\right]=E_{1,1}(-\frac{Et^{\alpha }}{\eta })$$

Under the assumptions of this paper, the analytical solution Equation (7) is:

(19) $$\tau (t)=\int_{0}^{\Delta t}\mu_{d}\nabla {\bar{U}e}^{-\frac{Et^{\alpha }}{\eta }}dt\;\nabla \bar{U}=\frac{1}{\Delta t}\int_{0}^{\Delta t}\nabla Udt$$

where Δt is the time of the fluid during half the length of the flow through the capillary. Based on Jasak’s definition of the FVM discretization operator in OpenFOAM [39], Equation (10) after semidiscretization is:

(20) $$A_{P}U_{P}^{n+1}+\sum A_{N}U_{N}^{n+1}=-\nabla P+S_{P}^{n}+g\cdot h\nabla \rho_{P}^{n+1}+\sigma \kappa \nabla \alpha_{P}^{n+1}+\nabla \cdot \tau_{p}^{n}$$

The $\nabla \cdot \tau_{p}$ in Equation (19) is treated explicitly according to the analytical solution that we obtained. Therefore, based on the interpolation format of Rhie-Chowthe, the surface velocity $U_{p}^{n+1}$ that we need to calculate is:

(21) $$U_{P}^{n+1}=\mathrm{Hby}A_{P}^{n+1}-\frac{1}{A_{P}}\left(\nabla p_{\mathrm{rgh},P}^{n+1}+g\cdot h\nabla \rho_{p}^{n+1}-\sigma \kappa \nabla \alpha_{P}^{n+1}-\nabla \cdot \tau_{P}\right)\;\mathrm{Hby}A_{P}^{n+1}=\frac{1}{A_{P}}\left(-\sum A_{N}U_{N}^{n+1}+S_{P}^{n}\right)\;\nabla p_{rgh}=\nabla p-g\cdot h\nabla \rho -\rho g$$

The pressure correction equation is:

(22) $$\nabla \cdot \left(\frac{1}{A}\nabla p_{\mathrm{rgh}}^{n+1}\right)=\nabla \cdot \left(HbyA^{n+1}+\frac{1}{A}\left(\sigma \kappa \nabla \alpha^{n+1}-g\cdot h\nabla \rho^{n+1}\nabla \cdot \tau_{p}\right)\right)$$

## 3. Result and Analysis

Without loss of generality, the involved variable parameters are given as follows: the fractional-order α is 0.1, 0.2, 0.3, 0.5; the relaxation time λ is 0.1 s; the polymer solution flow rate is $2\times 10^{-5}m/s$; and the fluid parameters are shown in Table 1. Because the polymer is completely dissolved in water, the density of the polymer and water are equal. Before being dissolved in water, the polymer is solid, and its viscosity and volume fraction cannot be measured. Therefore, the viscosity of the polymer is an approximate viscosity, which calculates an equation such as:

(23) $$\mu_{p}=\frac{\mu_{d}\left(M_{w}+M_{p}\right)-\mu_{w}M_{w}}{M_{p}}$$

### 3.1. Effect of α on Displacement Efficiency

**Figure 5:**
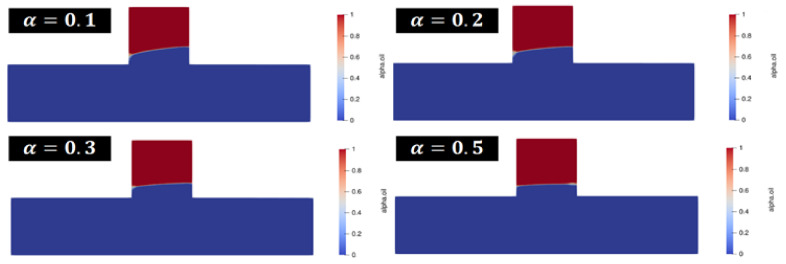
The results of simulation for the polymer flooding.

**Figure 6:**
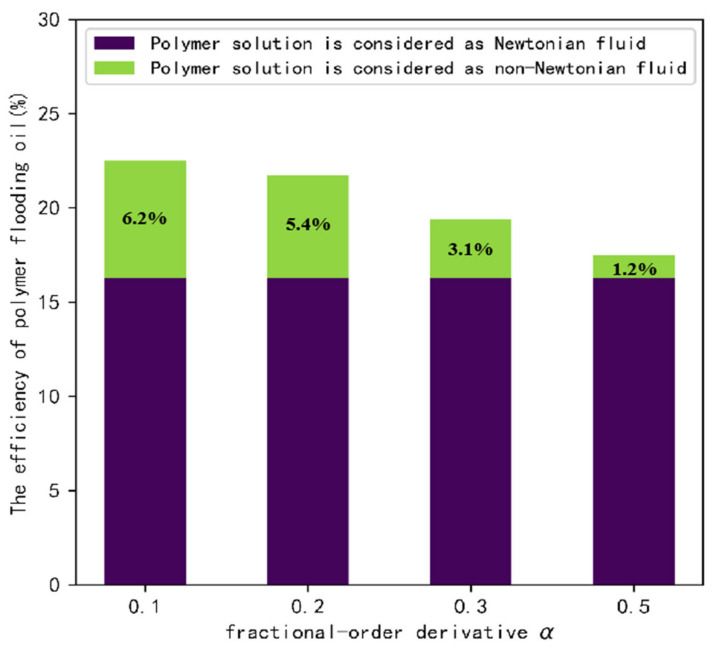
The effect of fractional-order derivative α on the efficiency of polymer flooding oil.

Figure 5 shows the results of flooding the remaining oil at the dead end with a polymer solution of the different fractional-order derivative α. The oil-water interface formed after the polymer solution with fractional-order derivative α=0.5 intrudes into the dead, and has a low inclination and a rectangular shape. With the gradual decrease of α, the inclination of the oil-water interface becomes large and has a trapezoid shape. Figure 6 shows the efficiency of polymer flooding oil with a different fractional-order derivative α. The purple part of the graph shows the efficiency of polymer flooding oil obtained from the simulation when the polymer solution is considered a Newtonian fluid. The green part indicates the contribution of the fractional-order velocity field to the efficiency of polymer flooding oil. When a is smaller, the contribution of the fractional-order velocity field is larger and the efficiency of polymer flooding oil obtained is higher; in addition, the growth rate is exponential, which is consistent with the form of the fractional-order analytical solution obtained.

### 3.2. Effect of α on Elastic Perturbation in the Dead End

**Figure 7:**
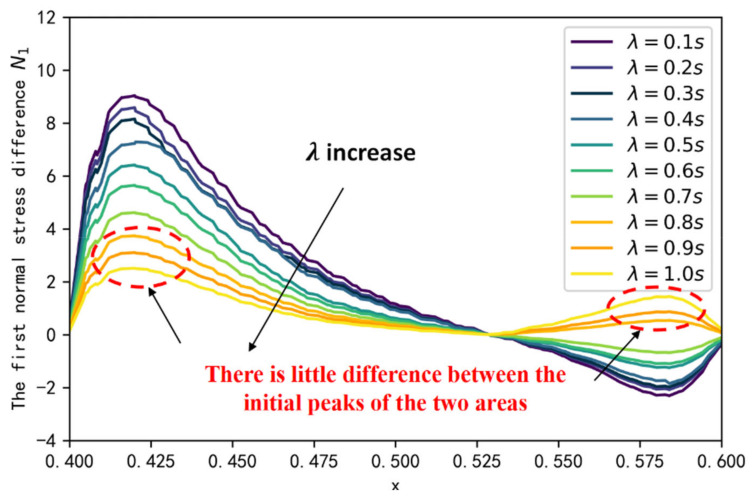
The first normal stress difference at the oil-water interface in the dead end.

**Figure 8:**
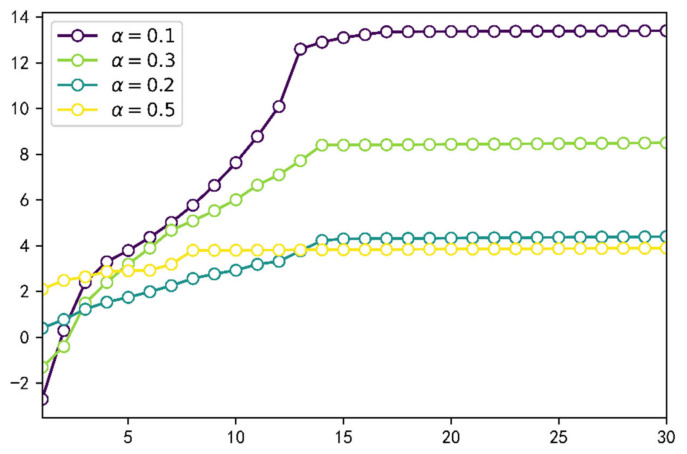
The peak variable of the elastic perturbation area 2.

The first normal stress differences can indicate the elastic behavior of the fluid during the flow, which is one of the main differences between a viscoelastic fluid and a Newtonian fluid. We extracted the first normal stress difference at the oil-water interface in the dead end at t = 1 s. As illustrated in Figure 7, there are two elastic perturbation regions, and the presence of a maximum value in the elastic perturbation region, which is strongly dependent on the parameter α; i.e., the smaller parameter α is, the bigger peak of the elastic perturbation region is. Viewing the $N_{1}$ and polymer flooding oil processes at the dead end in conjunction, the polymer solution gradually invades into the dead end from the left side to the right side, and the elastic wave is also transmitted from the left side to the right side. Figure 8 illustrates the peak variable of the elastic perturbation area 2 during t = 1 s to t = 30 s. Results indicate that, for each fractional-order derivative α, the peak value maximum of the elastic perturbation area 2 is monotonically increasing; thus, the smaller parameter α is, the larger the increase rate is. This phenomenon indicates that the higher peak of the perturbation area 1, the larger the initial range of polymer invasion into the blind end, the more elastic wave energy elastic perturbation area 2 receives, and the higher efficiency of polymer flooding oil.

### 3.3. Effect of λ on Displacement Efficiency

**Figure 9:**
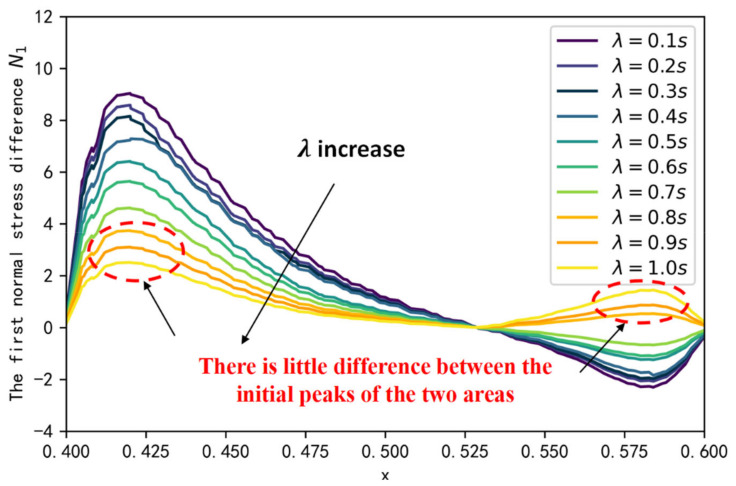
The first normal stress at the oil-water interface for different λ.

**Figure 10:**
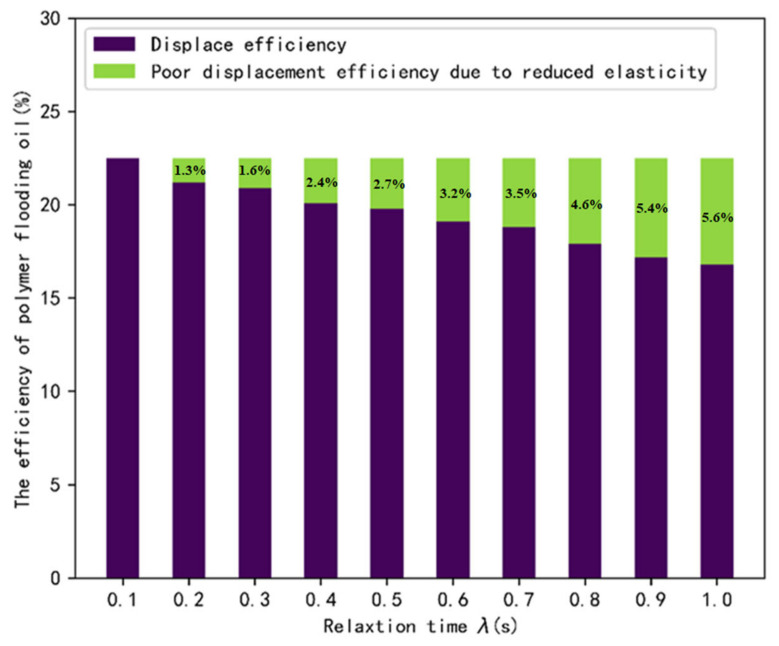
The displacement efficiency for different λ.

Relaxation time λ can indicate the ratio of the elastic and viscous portions within the fluid. According to Section 3.1, we chose a polymer solution with fractional-order α=0.1 as the basis of our study, and investigated the effect on displacement efficiency by changing the relaxation time λ. Figure 9 and Figure 10 illustrate the effect of λ on the first normal stress difference at the oil-water interface in the dead end at t = 1 s, and the effect of λ on displacement efficiency. It is found that the peak of the elastic perturbation area 1 gradually decreases with increasing λ, indicating that the elasticity of the fluid is the main factor affecting the first normal stress difference. As λ approaches 1, a phenomenon similar to that for α= 0.5 occurs, with a lower peak in the elastic perturbation area 1 and a smaller difference between the peak in the elastic perturbation area 2. This indicates that there is a no larger transfer of elastic wave energy between the left and right sides of the blind end with the flow of the polymer solution. Therefore, after the polymer solution invades from the left side of the blind end, it cannot gradually invade deeper into the blind end through the change of the normal corresponding force difference between the two sides of the blind end, resulting in the reduction of displacement efficiency.

## 4. Conclusions

This paper studies the unsteady process of the viscoelastic fluid flooding oil in the dead end. The fractional derivative is introduced in Maxwell’s constitutive model. Analytic solutions of the fractional order Maxwell model were computed and implanted into the N-S equations by showing the forces. The analytical solution of the fractional-order Maxwell model is calculated, and a semi-analytical solution model for viscoelastic fluid flow is developed by making the viscoelastic stresses explicit. The process of polymer flooding is simulated by performing a secondary development of the interFoam incompressible two-phase flow solver in OpenFOAM. Some useful conclusions are drawn from the simulation results:

1. The viscoelastic fluid is significantly more effective in displacing the remaining oil in the dead end than the Newtonian fluid;
2. The perturbed region of viscoelastic fluid within the blind end can be divided into two, which gradually invade deeper into the dead end through the elastic wave transmission between the two areas;
3. The smaller the fractional order derivative a, the greater the fluid viscoelasticity and the higher the oil displacement efficiency;
4. The smaller the fractional order derivative a, the larger the first normal stress difference peak in the elastic perturbation region 1, the greater the fluid viscoelasticity, and the higher the oil displacement efficiency;
5. The relaxation time of the fluid has a significant effect on the viscoelasticity of the fluid, and when the relaxation time is close to 1 s, the flow characteristics of the fluid gradually change from viscoelastic to pure viscous fluid.

## Figures and Tables

**Table 1:** Fluid parameters of oil and polymer solution.

	Oil	Displacement Fluid ($\mu_{d}\;=\;20\;mPa\cdot s$)		
		Water	Polymer ($M_{p}\;=\;1200\;mg/L$)	
Density (kg/m^3^)	860	1000		1000
Viscosity (mPa·s)	9	1		4 × 104
Interfacial tenso (mN/m)		5		
